# Reduction of *Escherichia Coli* Using Metal Plates with the Influenced of Applied Low Current and Physical Barrier of Filter Layers

**DOI:** 10.3390/ijerph16203887

**Published:** 2019-10-14

**Authors:** Michael Versoza, Wonseok Jung, Mona Loraine Barabad, Sangwon Ko, Minjeong Kim, Duckshin Park

**Affiliations:** 1 Transportation Environmental Research Team, Korea Railroad Research Institute, Uiwang City 16105, Korea; mikeverz23@krri.re.kr (M.V.); worship611@skku.edu (W.J.); mlmbarabad@gmail.com (M.L.B.); sko@krri.re.kr (S.K.); mjkim88@krri.re.kr (M.K.); 2 Railway System Engineering, University of Science and Technology, Daejeon City 34113, Korea; 3 Mechanical Engineering Department, Sungkyunkwan University, Suwon City 16419, Korea

**Keywords:** Contact-Killing, Metal-Plates, electricity, Filter-Layers, bacteria, virus

## Abstract

Although metal contact is known to reduce bacterial growth, the effects of physical barriers and electricity need further investigation. This study examined the bacteria-reducing properties of copper and stainless-steel metal plates with an added electrical current and up to three filter layers on the growth of *Escherichia coli* (bacteria) and MS2 bacteriophages (virus). When used with a stainless-steel plate, electricity increased bacteria reduction by 39.5 ± 2.30% in comparison with no electricity added, whereas a three-layer physical barrier decreased its efficiency. Copper also reduced the growth of bacteria, by 58.2 ± 8.23%, and the addition of electricity reduced it further (79.5 ± 2.34%). Bacteriophages were also affected by the metal contact. Further experiments showed that MS2 was also reduced by copper, to 82.9 ± 4.5% after 24 h at 37 °C.

## 1. Introduction

Copper metal surfaces kill different species of bacteria efficiently (Grass *et al*., 2011). Prolonged contact leads to damage to cell membranes, which are overwhelmed by intracellular copper, reducing bacterial growth [1,2]. Contact killing is usually rapid, and no bacteria fully resistant to copper have been discovered [3].

The biocidal properties of copper metal surfaces provide an easy way to counter pathogen spread via human carriers. Egyptian writing from 2600–2200 BC indicated that copper was used to treat chest wounds and drinking water [4]. In developing countries, copper is used for water treatment because of its low cost [5]. Doorknobs and other touched surfaces are potential sources of nosocomial infection, and some studies have reported that materials made from brass and bronze prevent the spread of microbes in hospitals [6]. Marais et al. (2010) [7] observed an overall 71% reduction in bacteria in a room where items were covered with copper sheets. Depner et al. (2016) [8] conducted a study in commercial poultry hatcheries and found that mesophilic microorganisms, fungi, and yeasts were absent when the samples were exposed to copper surfaces compared with stainless-steel. Stainless-steel is commonly used in healthcare facilities due to its corrosion resistance and dirt-free appearance, but it has no antibacterial properties [9].

One mechanism of copper toxicity is the change between its cuprous [Cu(I)] and cupric [Cu(II)] oxidation states. This involves a Fenton-type reaction that generates reactive hydroxyl radicals. The copper competes with other metal ions that play important roles in protein binding sites [10] and displaces iron from iron-sulfur clusters [11].

The application of a direct current has also been used to kill bacteria [12,13,14]. Electricity is thought to damage cell membranes through the irreversible loss of the semi-permeable barrier function [15]. A direct current killed *Escherichia coli* in liquid suspension, which depended on the strength of the current [16,17], although the liquid medium might also have affected the efficiency of electricity in these studies. By contrast, Zituni et al. (2014) [18] found that a low direct current with gold electrodes on solid agar had low efficiency, although they demonstrated that increasing the electrical field increased the membrane potential, thereby hindering proton translocation. This reduced both the pH and adenosine triphosphate (ATP) synthesis outside the cell membrane. An electrical field might be inefficient if the medium itself cannot transfer it to the target bacteria. This might also be affected by the type of electrode used in the experiments [19]. Only a few studies have examined the influence of electricity on the growth of bacteria, and the authors wanted to explore the phenomenon further.

The present experiment used copper plates to examine the effects of electricity and solid medium (agar) on *Escherichia coli* and viruses, as copper is a good conductor and is known to kill bacteria. To examine the effects of metal conduction copper was compared with stainless-steel (SS). Further, whether a physical barrier (simple cloth filters) influenced the efficiency of killing bacteria with copper and electricity was also investigated to examine ways to improve the elimination of bacteria and viruses in water samples and other media.

## 2. Material and Methods

### 2.1. Bacterial and Virus Preparations

This experiment used F-specific RNA bacteriophages (*E*. *coli* MS2 bacteriophages; ATCC 15597-B1, Manassas, VA, USA) for plaque counting. MS2 stock solution was prepared in accordance with the product sheet from ATCC. The stock solution (1000 μL) was frozen at −75 °C, and some stock solution was incubated with the host to check its viability. Then, 1-μL aliquots of MS2 stock solution were added to 50 mL sterilized deionized water, and diluted by a factor of three. The incubated host was then added. *Escherichia coli* (C3000, ATCC 15597) was cultured in accordance with the product sheet from ATCC within 24 h. A stock solution of *E*. *coli* was mixed with 40 mL of sterilized tryptic soy broth (TSB) and shaken at 150–200 rpm at 37 °C in an incubator for 6–12 h. Then, 0.3 mL of host solution, 0.1 mL of bacteriophages, and 24 mL of soft agar (30 g TSB and 7.7 g agar in 1 L distilled water) were mixed and poured into Petri dishes.

### 2.2. Metal Plates, Filter and Applied Current

The experiment used stainless-steel (SS304) and copper (Cu-99.9%) plates (D&S Tech, South Korea) designed to fit in a 150-mm-diameter Petri dish with four external anchors to attach the cables from a low-frequency device generator (OTS H-306; Hanil Meditech Probe, South Korea) operating at 10 Hz for 50 min. In addition, 0–3 layers of filter paper were placed above the copper plates. Then, 0.3 mL of the *E*. *coli* and 0.1 mL of MS2 bacteriophages were used to determine the number of plaque-forming units (PFU) of the cultured host. Finally, 29 mL of tryptic soy agar (TSA) was added to the Petri dish and incubated at 37 °C for 12–18 h. 

### 2.3. Efficiency Reduction Calculations

**Figure 1:**
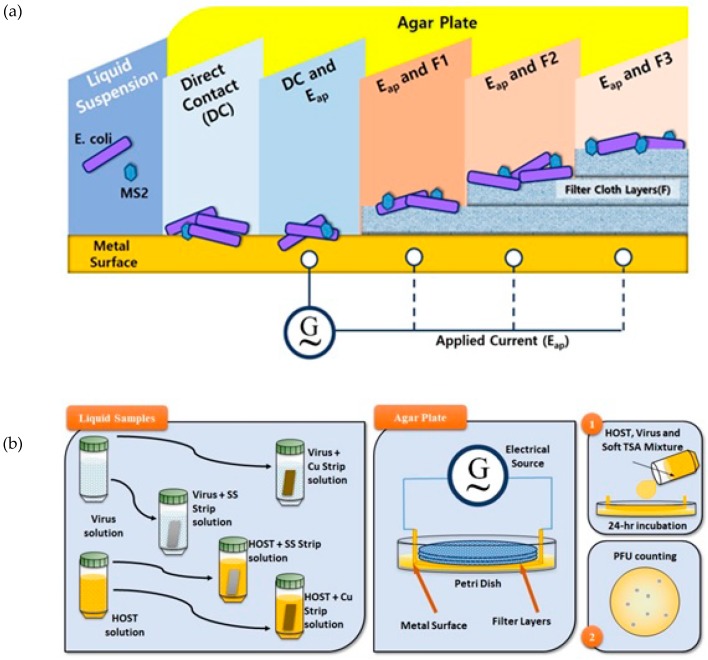
The conceptual framework of the study (**a**). All experiments with applied electricity were done on agar plate while the liquid samples were incubated first in liquid solutions before placing in agar plates. Liquid samples and Agar plates are also presented here in detail (**b**). All samples were incubated in 24 h and PFU counting were done afterwards.

Figure 1a outlines the experimental design. The Petri dishes with 30–300 PFUs were used to compare the metal-treated and untreated samples and quantify the effects. All plates were examined, and the number of colonies in PFUs/mL was calculated as follows:

(1) $$N_{\left(\frac{CFU}{mL}\right)}=\frac{\mathrm{PFU}}{\alpha }\times \frac{1}{d_{i}}$$

where PFU is the total plaque count in the Petri dish, *α* is the volume of virus added (mL), and *d_i_* is the dilution factor of the virus. After counting the plaques in each plate, the concentrations were expressed in logarithmic form (base 10). The reduction efficiency (%) was calculated as follows:

(2) (%) Reduction Efficiency={1−(NTreatedNControl)}×100

where *N_Treated_* is the metal-treated samples, and *N_control_* is the control. The log_10_ difference in *N* was calculated as follows:

(3) $$Log\;Diff_{\left[Log_{10}\left(N\right)\right]}=Log_{10}\left(N_{control}\right)-Log_{10}\left(N_{treated}\right)$$

where *Log Diff* is the difference between the logarithmic values of the control plaques and the plaques with metal plates and filter layers. The differences between treatments with and without electricity were also calculated. All calculations were based on our previous study [20]. All experiments were repeated multiple times, and three to four experiments with consistent data were used to calculate the geometric means and standard deviations.

### 2.4. Liquid Samples with Metal Strips

This study also examined whether copper or stainless-steel metal strips influenced the growth of the model virus and its host (Figure 1b). In the experiments using agar plates, both the host and virus were present. The presence of metal might also affect the behavior of the virus. To address this, *E*. *coli* and MS2 were prepared separately under the same conditions as above (30 mL total solution in 50 mL polypropylene tubes) with a rectangular metal strip (−0.2 g) and incubated at 37 °C and 150–200 rpm. The MS2 was incubated without its host to determine the longevity of the virus in water samples with and without the metal strips. These were prepared by adding stock solution (1 µL) to 50 mL of distilled water and separating the mixture into three parts (one each for the control and each metal). The *E*. *coli* was also prepared with stock solution and separated into three parts. After 7 and 24 h, 1 mL of liquid was withdrawn. For the metal-treated and control *E*. *coli* liquid samples, fresh virus stock solution (0.1 mL diluted to 50 mL with deionized water) was ready for mixing in a Petri dish and the same as for metal-treated and control MS2 liquid samples after incubating the host for 6–7 h at 37 °C and 150–200 rpm. This experiment was done in triplicate.

## 3. Results

### 3.1. PFU Difference with the Influence of Filter Layers and Electricity.

The log differences between the control and treated samples were determined for all experimental conditions (Table 1). The log difference was higher in the presence of a Cu plate only (Cu-0, no filters) than with a SS plate only (SS-0, no filters) (0.379 ± 0.05 versus 0.137 ±0.02 log_10_ PFU/mL), indicating greater inactivation with copper. By contrast, with three filter layers, the log difference was higher with SS-0 than with Cu-0 (0.361 ± 0.02 vs. 0.308 ± 0.10 log_10_ PFU/mL). This indicated that the *E*. *coli* was affected more by the presence of Cu, but the bacterial growth was affected less as the filter layers increased.

When current was applied to the metal plates (SS-Eap and Cu-Eap), the log difference was higher compared with no voltage (SS-0 and Cu-0), increasing to 0.476 ± 0.14 and 0.688 ± 0.01 log_10_ PFU/mL for the SS and Cu plates, respectively, without added filters. With three filter layers, the log difference decreased from 0.572 ± 0.02 with zero filters with the Cu plate to 0.368 ± 0.13 and 0.242 ± 0.17 log_10_ PFU/mL with one, and three filter layers, respectively; with the SS plate, it decreased from 0.353 ± 0.14 with zero to 0.348 ± 0.02 and 0.339 ± 0.04 log_10_ PFU/mL with one and three filter layers, respectively.

### 3.2. Influenced on Filter Layers

**Figure 2:**
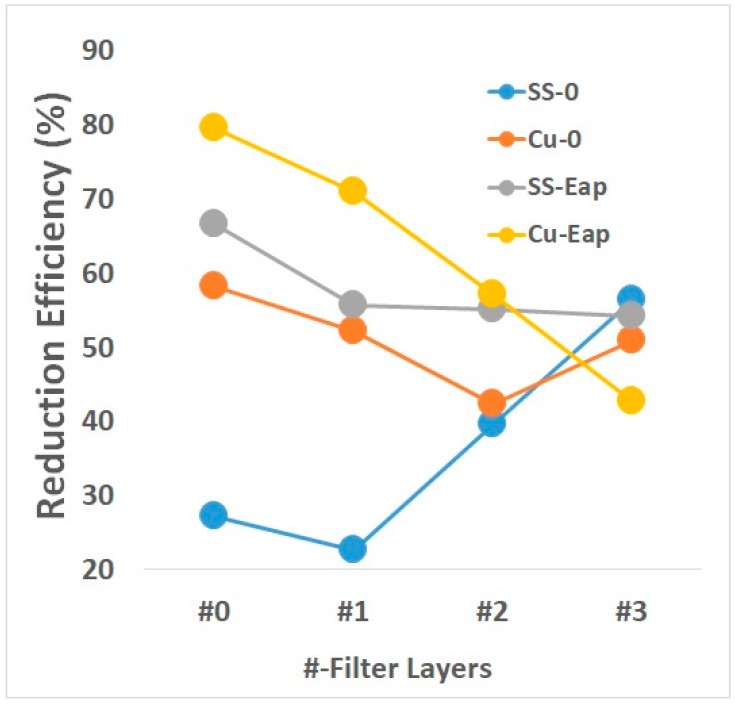
Inactivation efficiency of the non-applied and applied current experiments.

The filter layers reduced the effects of the metal plates on the host bacteria. With SS-0, the inactivation was directly proportional to the increasing number of filter layers, at 27.1 ± 6.00, 22.6 ± 6.21, 39.6 ± 3.50, and 56.4 ± 8.61% for 0 to 3 layers, respectively (Figure 2). By contrast, the inactivation with Cu-0 was indirectly proportional to the number of filter layers, at 58.2 ± 8.23, 52.2 ± 5.42, 42.4 ± 4.53, and 50.8 ± 5.43%, respectively.

With electricity, the inactivation efficiency of SS-Eap was 66.6 ± 4.51, 55.7 ± 5.53, 55.1 ± 10.32, and 54.2 ± 3.2% as the number of filter layers increased, whereas the values for Cu-Eap were 79.5 ± 2.34, 71.0 ± 8.92, 57.1 ± 2.36, and 42.8 ± 1.32%, which surpassed the values for stainless-steel using a current with fewer filter layers. Both metal plates had roughly the same inactivation efficiency with three filter layers.

### 3.3. Differences of Applied Current

**Figure 3:**
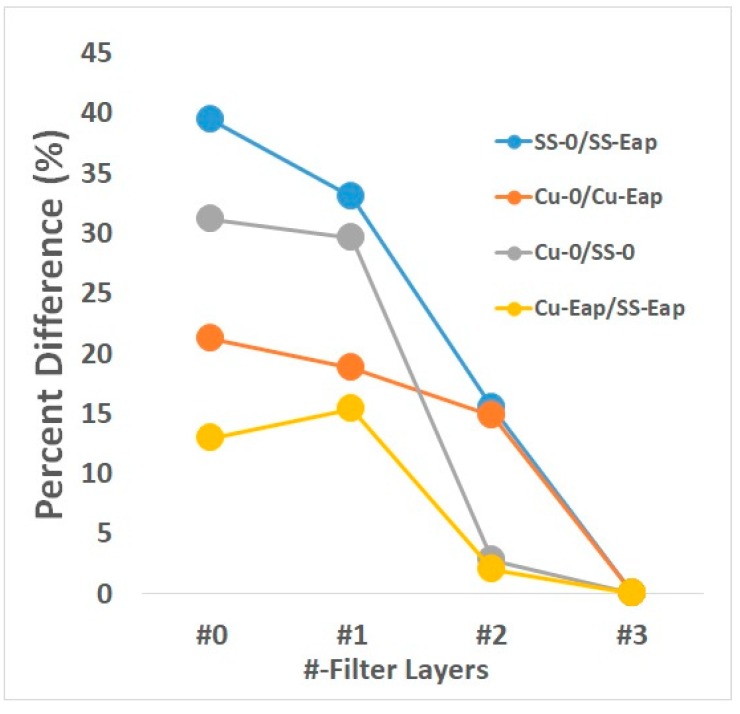
The percentage difference between the applied and non-applied current of metal plates with the influence of filter cloth layers.

Figure 3 shows the percentage difference (*Diff_%_*) between the stainless-steel (SS-0/SS-Eap) and copper (Cu-0/Cu-Eap) metal plates without (Cu-0/SS-0) and with (Cu-Eap/SS-Eap) current. Comparing both metals without a current, Cu-0/SS-0 had a difference of 31.14 ± 1.60%. This decreased to 29.57 ± 1.90% with one and 2.78 ± 0.21% with two filter layers. With an applied current, the difference between metals was 12.92 ± 5.20%. The difference increased slightly to 15.32 ± 3.10% with one filter layer and fell to 2.02 ± 0.09% with two layers.

Comparing each metal with and without a current, SS-0/SS-Eap had a difference of 39.5 ± 2.30%, which decreased to 33.1 ± 4.50% (one filter layer) and 15.5 ± 1.70% (two filter layers). Cu-0/Cu-Eap had a difference of 21.3 ± 3.20%, which decreased to 18.8 ± 2.80% (one filter layer) and 14.8 ± 1.20% (two filter layers). The plates of both metals had roughly the same inactivation efficiency with three filter layers. The difference was scored as 0%.

### 3.4. Metal Strips in Liquid Samples

**Figure 4:**
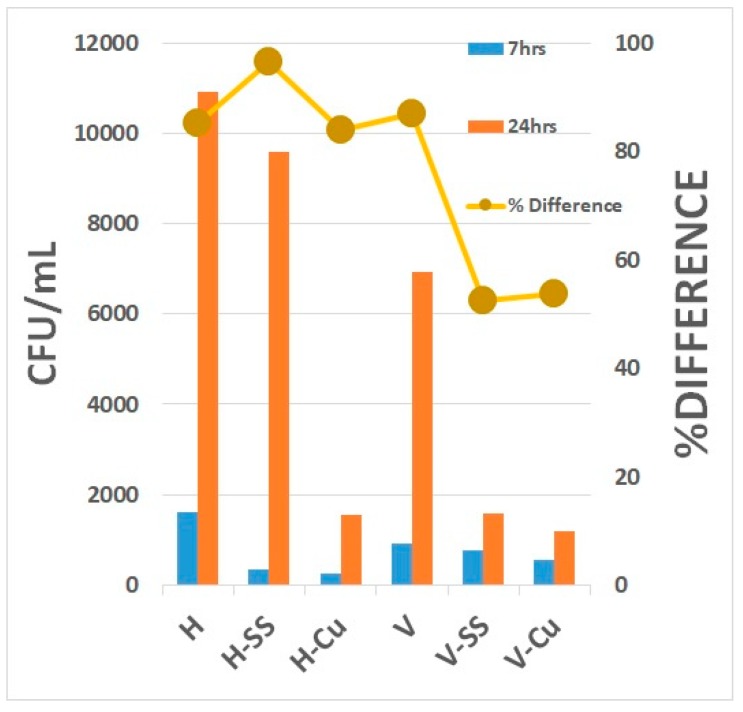
Colony-forming unit (PFU/mL) between incubation intervals of 7-hrs and 24hrs. Conditions are H: *E. coli,* H-SS: with stainless-steel, H-Cu: with copper; and V: MS2 Bacteriophages; V-SS: with stainless steel, and V-Cu: with copper.

To determine whether the metal strips influenced the behavior of MS2, the metal strips were added during the incubation of MS2 and *E*. *coli* separately. After incubating 1500 PFU/mL for 7 h, the counts for the *E*. *coli* (H) and MS2 (V) controls were 10,931 ± 60.07 and 6941 ± 52.3 PFU/mL, respectively. With the metal strips, the values were lower, at 9581 ± 28.9 (H-SS) and 1586 ± 62.0 PFU/mL (V-SS), respectively, for stainless-steel and 1536 ± 17.45 (H-Cu) and 1188 ± 30.2 PFU/mL (V-Cu) for copper (Figure 4).

**Figure 5:**
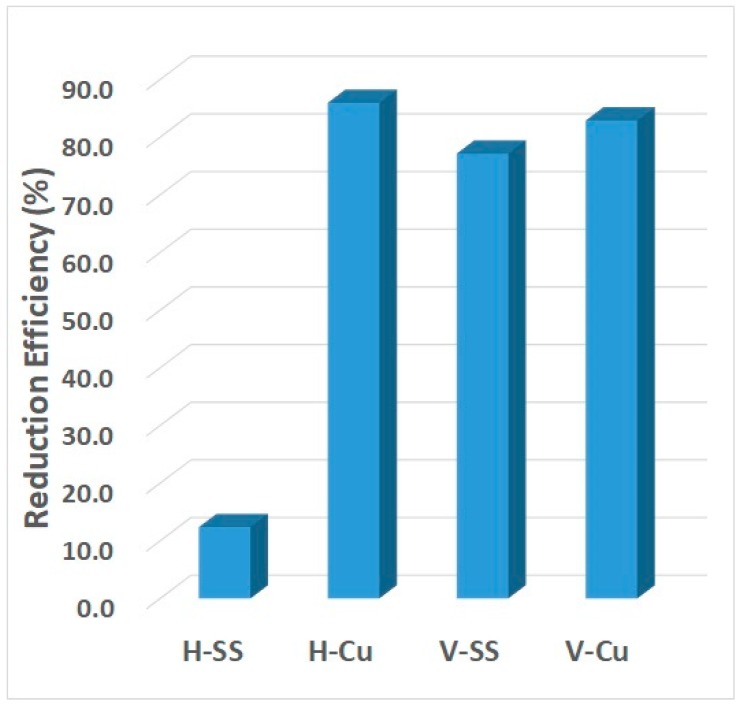
Reduction efficiency of liquid samples with metal strips. H-SS: with stainless steel, H-Cu: with copper; and V-SS: with stainless steel, and V-Cu: with copper.

After incubation for 24 h, the host levels increased in all samples, with a percentage difference (between 7 and 24 h) of 85.2 ± 2.5, 96.5 ± 10.2, and 83.9 ± 15.2% for the control, stainless-steel strip, and copper strip, respectively (Table 2). The MS2 count difference increased to 87.0 ± 3.2% for the control, but decreased with both the stainless-steel (52.5 ± 5.1%) and copper (53.7 ± 3.4%) strips. After incubating the liquid samples for 24 h, there was a significant increase in the reduction efficiency of both *E*. *coli* and MS2 bacteriophages, as shown by comparing the control and metal-treated samples. With the copper metal strips, the host and virus samples were reduced to 86.0 ± 6.5% and 82.9 ± 4.5%, respectively, whereas with stainless-steel metal strips, the virus was reduced to 77.2 ± 2.1% and the *E*. *coli* to 12.4 ± 3.2% (Figure 5).

## 4. Discussion

Copper is essential in biological systems, but higher concentrations can be toxic and lead to the generation of hydrogen radicals that can damage cellular [21,22,23,24]. The reaction of dihydrogen peroxide with superoxide occurs at a constant rate (Equation (1)), and is accelerated in the presence of copper or iron. Cu (II) is reduced by superoxide (Equation (2)) and re-oxidized by dihydrogen peroxide (Equation (3)), which results in the production of hydrogen radicals.

(4) $$H_{2}O_{2}+O_{2}^{-}\to O_{2}+OH^{-}+OH^{•}$$

(5) $$Cu\left(II\right)+O_{2}^{-}\to Cu\left(I\right)+O_{2}$$

(6) $$Cu\left(I\right)+H_{2}O_{2}\to Cu\left(II\right)+O_{2}+OH^{-}+OH^{•}$$

Hydrogen radicals are highly reactive oxidants with a half-life of 1 nanosecond in aqueous solution, and they can damage biomolecules in cells [25]. Copper can denature *E*. *coli* DNA [2], as can superoxide from the generation of hydroxyl radicals [11,26].

Copper can reduce the growth of bacteria and viruses, as demonstrated in this study with *E*. *coli* and MS2 bacteriophages. Copper metal surfaces can potentially kill pathogens. It was found that a 24-h exposure to copper resulted in greater reductions. A greater reduction in the growth of *E*. *coli* was also observed when a voltage was applied to the copper, as shown in Figure 2 and Figure 5. However, even with an applied current, no significant change occurred when three filter layers were used. Under this condition, stainless-steel and copper had roughly the same inactivation efficiency. SS-0 outperformed Cu-0 when a current was applied, but not when filter layers were added.

In contact killing, there was a large difference as increasing filter layers were added. Without a current, stainless-steel (SS-0) had lower efficiency, demonstrating that stainless-steel is not ideal for inactivating *E*. *coli*. With increasing filter layers, the bacteria and viruses had no contact with the metal, resulting in less change in the phages in the Petri dishes. Similarly, the physical barrier of the three filter layers eliminated the effects of copper and electricity on growth. The inactivation efficiency was also reflected in the PFU differences because the physical barrier prevented contact between *E*. *coli* and the metal surface. Stainless-steel alone did not affect the growth of bacteria, and the physical barrier of filters also prevented the virus and its host from contacting the metal, lowering the inactivation efficiency.

The possibility that MS2 might also be affected by the metal plates was investigated by separating it from its host bacteria. Using liquid samples, the effects of 0.2-g metal strips on the virus after incubation for 7 or 24 h could be seen. The separate growth of the host bacteria and viruses was greater after 24 than after 7 h. MS2 bacteriophages incubated at 37 °C were still active after 24 h. The increase in MS2 growth might have been influenced by the use of a fresh host (6-h incubation), as the growth of phages adapts to factors such as temperature, growth phase, pH [27], or the genomics of its host bacteria [28]. In our study, both conditions influenced its growth because the growth of the control was significantly higher than that of the samples with the metal strips. The virus was reduced by more than 50% (based on time intervals) with both metals. Other studies have shown that the growth of different viruses was reduced by copper or silver. Copper affected the growth of influenza A virus [29] after 6 h, but not other bacteriophages [30]. Copper exhibited higher inactivation efficiency for MS2 bacteriophages in our study than in another study using different copper concentrations [31]. The mechanism by which copper affects in MS2 on a genomic scale remains to be determined.

The host bacteria were reduced less in the liquid samples with stainless-steel than in those with copper strips. In the experiments incubating the host bacteria samples directly on Petri dishes and in liquid samples, copper metal outperformed stainless-steel under both conditions Although the effect of this metal in both liquid and agar is promising, further studies are needed to address the application of these results to strategies for reducing the effects of viruses and bacteria on human health in clinical practice.

## 5. Conclusions

This study investigated the effects of metal plates on contact killing of bacteria and viruses and the influences of electricity and physical barriers on those effects. Electricity increased the reduction in bacterial growth, but this effect was reduced by a physical barrier. Metals also reduced the growth of viruses in liquid samples.

## Figures and Tables

**Table 1:** The difference of colony forming units (at Log base 10) between the blank samples and the treated samples with metal plates and filter layers.

Log Difference				
Treatment	Filter			
	#0	#1	#2	#3
SS-0	0.137 ± 0.02	0.111 ± 0.09	0.219 ± 0.03	0.361 ± 0.02
Cu-0	0.379 ± 0.05	0.354 ± 0.05	0.239 ± 0.09	0.308 ± 0.10
SS-Eap	0.476 ± 0.14	0.353 ± 0.14	0.348 ± 0.02	0.339 ± 0.04
Cu-Eap	0.688 ± 0.01	0.572 ± 0.02	0.368 ± 0.13	0.242 ± 0.17

* Note: Stainless steel and copper with NO electricity added (SS-0, Cu-0) and with added electricity (SS-Eap, Cu-Eap).

**Table 2:** Colony-forming unit (PFU/mL) of different condition at different incubation time.

Condition	Incubation Time		% Difference
	7 h	24 h	
*E. coli* (Control)	1621.9 ± 2.3	10935.1 ± 32.4	85.2 ± 2.3
*E. coli* + SS	336.9 ± 10.2	9580.9 ± 25.1	96.5 ± 2.5
*E. coli* + Cu	247.8 ± 15.2	1535.9 ± 35.6	83.9 ± 2.6
*MS2* (Control)	905.6 ± 3.2	6940.9 ± 20.3	87.0 ± 3.2
*MS2* + SS	753.1 ± 45.2	1586.1 ± 13.2	52.5 ± 5.1
*MS2* + Cu	550.3 ± 55.2	1188.0 ± 65.2	53.7 ± 6.5

* % Difference: The difference between 24-h and 7-h incubation time at percentile.
